# Investigating social determinants of child health and their implications in reducing pediatric traumatic injury: A framework and 17-year retrospective case-control study protocol

**DOI:** 10.1371/journal.pone.0294734

**Published:** 2023-11-27

**Authors:** Hunter Goodon, Justin Gawaziuk, Brenda Comaskey, Tracie O. Afifi, Dan Château, Marni Brownell, Jitender Sareen, Cora Morgan, Sarvesh Logsetty, Rae Spiwak

**Affiliations:** 1 Department of Surgery, Max Rady College of Medicine, University of Manitoba, Winnipeg, Manitoba, Canada; 2 Department of Community Health Sciences, Max Rady College of Medicine, University of Manitoba, Winnipeg, Manitoba, Canada; 3 Department of Psychiatry, Max Rady College of Medicine, University of Manitoba, Winnipeg, Manitoba, Canada; 4 ANU College of Health and Medicine, Australian National University, Canberra, Australia; 5 Data Analysis in Population Health Hub, National Centre for Epidemiology and Population Health, Canberra, Australia; 6 Manitoba Centre for Health Policy, Max Rady College of Medicine, University of Manitoba, Winnipeg, Manitoba, Canada; 7 Department of Psychology, University of Manitoba, Winnipeg, Manitoba, Canada; 8 Assembly of Manitoba Chiefs, Winnipeg, Manitoba, Canada; 9 Firefighters Burn Unit, Health Sciences Centre, Winnipeg, Manitoba, Canada; Duke University Medical Center: Duke University Hospital, UNITED STATES

## Abstract

**Introduction:**

Traumatic physical injuries are the number one cause of hospitalization and death among children in Canada. The majority of these injuries are preventable. The burden from injury can be reduced through prevention programs tailored to at-risk groups, however, existing research does not provide a strong explanation of how social factors influence a child’s risk of injury. We propose a theoretical framework to better understand social factors and injury in children and will examine the association between these social factors and physical traumatic injury in children using large population-wide data.

**Methods and analysis:**

We will examine data from 11,000 children hospitalized for traumatic physical injury and 55,000 matched uninjured children by linking longitudinal administrative and clinical data contained at the Manitoba Centre for Health Policy. We will examine 14 social determinants of child health measures from our theoretical framework, including receipt of income assistance, rural/urban status, socioeconomic status, children in care, child mental disorder, and parental factors (involvement with criminal justice system, education, social housing, immigration status, high residential mobility, mother’s age at first birth, maternal Axis I mental disorder, maternal Axis II mental disorder and maternal physical disorder) to identify groups and periods of time when children are at greatest risk for traumatic physical injury. A conditional multivariable logistic regression model will be calculated (including all social determinant measures) to determine odds ratios and adjusted odds ratios (95% confidence interval) for cases (injured) and controls (non-injured).

**Ethics and dissemination:**

Health Information Privacy Committee (HIPC No. 2017/2018-75) and local ethics approval (H2018-123) were obtained. Once social measures have been identified through statistical modelling, we will determine how they fit into a Haddon matrix to identify appropriate areas for intervention. Knowing these risk factors will guide decision-makers and health policy.

## Introduction

Traumatic physical injuries are the primary cause of childhood mortality and one of the leading causes of hospitalization in children [1]. In Canada, over 16,000 children are hospitalized every year for injuries [2]. Injuries have lasting effects on a child’s mental and physical health and social outcomes [3, 4]. Over 90% of unintentional childhood injuries are preventable [2] and prevention programs fuelled by evidence are needed. The success and cost effectiveness of prevention programs can be improved by tailoring programs to specific targeted needs [5]. We propose a protocol to examine social determinants of children’s health (SDoCH) and identify targets for the development of child traumatic injury prevention programs. SDoCH are a broad category of social and economic factors that influence a child’s health [6] and typically include factors related to the child’s environment including housing, parental health and those that reflect social support for a child’s development (e.g., community resources and services). While recognized as measures of inequity and important influences on the life cycle of a child, [7, 8] SDoCH have been less utilized in the development of injury prevention programs [9]. The Canadian Pediatric Society (CPS) has advocated for a child injury prevention plan that uses a public health approach and includes the social determinants of health associated with injury risk [2]. However, the CPS also states research is lacking to support this approach due to difficulty understanding mechanisms, causes and risk factors associated with child injury [2]. We will address these limitations by presenting a SDoCH theoretical framework and conducting a novel whole-population study linking clinical and administrative data to measure which SDoCH are associated with child traumatic physical injury. This work will inform the development of child injury prevention programs targeting individuals and times of greatest vulnerability.

We will use whole-population administrative data from one Canadian province linked with clinical data from the provincial pediatric trauma registry to evaluate the effects of a broad range of SDoCH on traumatic injury risk in children. To achieve this goal, we will compare 14 measures of SDoCH in children hospitalized for traumatic injury with a matched uninjured control group from the general population. The injured children will be identified over a span of 17 years (2002–2019). Our hypothesis is that odds of injury will be greater for children with adverse SDoCH compared to children without these SDoCH. This proposal expands this field of research by: using linked clinical and whole-population administrative data; building on a theoretical framework that includes a biopsychosocial model of injury; [4] and development of a Haddon matrix of injury prevention [10] to enact clinical and policy change. Using linked data we can directly address gaps in the literature by examining whole-population pediatric injury data in a truly novel way, producing robust high-quality information. This information has important implications for children and families through informing decision-making and health policy for children at greatest risk for injury. This approach will help us to: 1) understand the association between 14 SDoCH and pediatric traumatic injury; and 2) identify specific entry points for prevention strategies to optimize uptake and efficiency.

This protocol is an evolution of our previous research in SDoCH and childhood burn injury. A study by our team looked at the role of 13 measures of SDoCH among pediatric burn survivors and found that there were marked differences between children that did and did not experience a burn injury. Specifically, children from low-income households, children in care (i.e., children involved in the child protection system), children from a family that received social assistance and children born to teen mothers were at greater risk of burn injury [10]. Findings from this work are being used to develop burn prevention education and resources for young mothers and other vulnerable groups. This work identified an additional factor that contributed to injury risk: physical disorder of the child’s mother. Other research has found that having a parent with physical disorders and hospitalizations may impact a child’s behaviour [11] and decrease the capacity for parents to supervise children. Research using these extensive measures of SDoCH has not been conducted on children with traumatic injury and we will fill this important knowledge and care gap using a large longitudinal whole-population sample.

### Theoretical framework

Our theoretical framework has unique strengths which will help us clarify the relationship between childhood traumatic injury and multifaceted measures of SDoCH in one of the largest longitudinal pediatric trauma populations studied to date. The proposed framework and research protocol builds on theoretical frameworks previously utilized by our team: a) *Biopsychosocial framework specific to pediatric injury* (S1 Table); and b) *Haddon matrix for injury prevention* (S2 Table). The biopsychosocial model allows us to create a conceptual framework of the various SDoCH. While identifying which SDoCH place children at greatest risk of traumatic injury is essential, identifying unique intervention points for prevention using the Haddon matrix is what extends the novelty of the protocol. The Haddon matrix is based on the premise that injuries result from harmful interactions between the individual, the agent, the physical environment and the socioeconomic environment [12]. (S2 Table). The Haddon matrix is a validated model used to identify sources and time periods of preventable injuries and potential intervention points [12]. Combined with the biopsychosocial model, the Haddon matrix will provide a unique perspective on SDoCH and their role in not only child traumatic injury risk but injury prevention and health promotion. The Haddon matrix can be applied to determine how human, physical environmental and socio-economic environmental factors fit into the ‘pre-injury,’ ‘injury’ and ‘post-injury’ time periods. Interventions will then be conceptualized to reflect the complex interaction between factors focusing on ‘pre-injury’ and ‘injury’ time periods. We will use this approach to identify intervention points for children who have experienced traumatic injury [13]. This theoretical framework is an expansion of previous work on children who experienced burn injury (S2 Table).

### Research aims and hypothesis

We aim to evaluate associations between a broad range of SDoCH and physical traumatic injury risk in children. Our study objective is to compare 14 SDoCH in children hospitalized with physical traumatic injury with a matched control group (without prior hospitalization for a traumatic injury) from the general population in Manitoba. We hypothesize that odds of injury will be greater for children with adverse SDoCH compared to children without adverse SDoCH.

## Methods and analysis

This study will take place in Manitoba, the central province in Canada, with a population of 1,390,249; 312,164 under the age of 18 [14]. The Manitoba Centre for Health Policy (MCHP) is a research centre within the University of Manitoba that houses a repository of de-identified provincial, universally-insured health services data and social services data, including social determinants of health, that enables linkage between clinical and social data [15]. We propose a 17-year case-control study matching cases (injured children) with controls (uninjured children from the general population) to examine SDoCH using anonymized administrative data housed at the MCHP repository linked with the clinical data in the HSC Winnipeg Children’s Hospital clinical trauma registry (“Pediatric Trauma Registry”). The total sample size for this study is approximately 66,000. We will calculate odds ratios (OR) and adjusted odds ratios (AOR) for the odds of injury associated with each SDoCH. We will use the identified factors to develop a Haddon matrix to guide creation of injury prevention programs.

### Definitions

#### Cases

Children (≤17 years of age) hospitalized with injuries identified from the Pediatric Trauma Registry over a seventeen-year period (2002–2019). The registry contains data from approximately 11,000 hospitalized children. The date of injury will be identified as the ‘index date.’

#### Controls

The cases (injured) will be matched 1:5 with controls from the general population (uninjured prior to the index date) based on sex, geographical region and age on the index date (55,000 matches).

#### Exposure risk factors (independent variables)

14 SDoCH measurable (at various time points/periods) using administrative data: Receipt of income assistance, rural/urban status, socioeconomic status, children in care, child mental disorder and parental factors (involvement with criminal justice system, education, social housing, immigration status, high residential mobility, mother’s age at first birth, Axis I mental disorder diagnosis, Axis II mental disorder diagnosis, maternal physical disorder).

#### Outcome (dependent variable)

Child hospitalized with an injury. We will look at the following subgroups: a) Major injury (Injury Severity Score (ISS) ≥12); b) Minor injury (ISS<12); and c) intentional (i.e., assault) and unintentional (i.e., accidental, non-assault) injuries. The standard measure of extent of injury is the Injury Severity Score (ISS). Based on identifying injuries in one of six body parts or regions, the ISS has been shown to correlate with mortality and morbidity [16]. In Canada, the definition of major trauma is an ISS≥12.

### Data sources

#### Pediatric trauma registry

This study will utilize linked data from the Pediatric Trauma Registry. All children in the province of Manitoba with a significant injury are treated at this hospital. Information recorded in this registry includes date of injury, demographic factors such as age and sex and injury related factors (etiology of the injury, e.g., motor vehicle collision; context of injury, e.g., recreational; admission to intensive care unit).

#### Manitoba Centre for Health Policy (MCHP)

MCHP is a research data centre within the University of Manitoba that houses a world-renowned repository of de-identified provincial health service data supplemented with a variety of other social services and public use datasets for the purposes of health-related research (over 90+ datasets) [15]. This rich, whole-population repository holds data over a 50-year period including health care usage (hospitalizations, emergency department use, ambulatory care and specialist physician visits, prescription drug utilization) and data on the following: vital statistics (birth and death records), Canada census, immigration, education, justice, use of income assistance and other social services including child protection and social housing.

#### Data linkage and matching

A password-protected copy of the Trauma Registry will be sent to the provincial ministry of health where the data will be anonymized (i.e., any identifying information removed) and a scrambled Personal Health Identification Number (sPHIN) attached. This anonymized data will then be sent to MCHP to be linked with the datasets using this sPHIN. Identifying information of participants will not be accessible to the authors prior to, during or after data collection and analysis. The administrative datasets for this study (S3 Table) include:

Pediatric Trauma Registry; Manitoba Health Registry; Hospital Separations Abstracts; Medical Claims (physician billings); Vital Statistics Mortality; Prosecutions Information Management System (PIMS); Employment / Income Assistance (SAMIN); Child and Family Services—Applications and Intake; Social Housing Tenant Management System (TMSI); and Enrollment, Marks, and Assessments (STS/ICAB). Immigration status is determined as previously described by Katz et al. [8] To the best of our knowledge, the linkage of the pediatric clinical injury registry with a whole-population data repository of this magnitude will be unique in Canada and the world.

### Social determinants of health to be evaluated

The SDoCH for this study are based on previous work by our research team, [10, 17] MCHP examinations of social determinants of health [18–20] and the literature. Based on work conducted by MCHP, there are 13 determinants that are measurable using the administrative datasets available. Our group has used these determinants to examine pediatric burn injury [10, 17]. Based on this previous work, we have added one additional factor to provide further investigation into the child’s environment: mother with a physical disorder. In Manitoba, almost 100% of children can be linked with their mother’s PHIN at birth; linkages to fathers are considerably lower, [15] making mothers’ health the best available marker for parents. Similarly, other parental measures (such as involvement in the justice system) may also reflect information pertaining to the child’s mother. SDoCH to be examined for this study are:

1. *Low-income household*. A child from a family living in a low income neighbourhood (lowest-income quintile), based on Canada census data.
2. *Rural status*: A child from a family residing in a postal code outside of a city with a population of >10,000 people, based on residential postal code at index date.
3. *Child from a family that has received income assistance*: A child from a family that has received financial income assistance from the Employment and Income Assistance Program (EIA) in Manitoba.
4. *Child with parent(s) involved in the justice system (as a victim*, *witness*, *or accused)*: A child with one or both parents involved in the justice system, based on data acquired via Prosecution Information and Scheduling Management (PRISM) developed by Manitoba Justice Prosecution Service.
5. *Child of parent(s) who did not graduate high school*: Child from a family where one or both parents did not complete high school, based on graduation status data from Manitoba Education.
6. *Child with parent(s) in social housing*: A child from a family who has lived in social housing managed by Manitoba Housing and Community Development.
7. *Child of parent(s) who immigrated*: A child of a parent from a native country that is not Canada, based on census data [8].
8. *Child from a family with high residential mobility*: A child from a family that moved residences three or more times within 10 person-years.
9. *Child of a teen mother*: A child of a mother who first gave birth at age 19 years or younger.
10. *Child with any mental health disorder*: The presence of an Axis I mental disorder measured using ICD codes (anxiety, depression, substance use disorder; See S4 Table). Both Axis I and Axis II disorders will be combined into one variable as we have observed that the prevalence of either of these diagnoses in children is relatively rare.
11. *Child of a mother with a major mental health disorder*: A child born to a mother with an Axis II mental disorder, based on ICD-9 diagnosis codes (See S4 Table). “Any Axis II Disorder” will combine Axis II diagnoses codes into one variable.
12. *Child of a mother with an Axis I mental disorder*: The presence of an Axis I mental disorder in the mother, measured using ICD codes (anxiety, depression, substance use disorder; See S4 Table). “Any Axis I Disorder” combines anxiety, depression and substance use into one variable.
13. *Child of a mother with a physical disorder*: Disorders of interest are based on previous work and will include: cardiovascular disease, cancer, chronic obstructive pulmonary disease, hypertension and diabetes [17]. (See S4 Table for diagnostic codes.) One or more hospitalizations pre-index date and/or one or more outpatient visits (physician billing) are considered a mental disorder or physical disorder.
14. *Child in care*: A child removed from family of origin and placed in the care of another adult due to concerns related to care, occurring at any time prior to the index date. This measure may be hypothesis-generating for future research in this area.

## Analytic strategy

SAS will be used for all analyses (Version 9.4, SAS Institute, Cary, NC, USA).

### Sample size and minimum detectable effect size

We have a fixed number of approximately 11,000 cases and an additional 55,000 matches for a total sample size of 66,000. We used prevalence estimates and identified effect sizes observed in previous work on SDoCH in burn injury [10] to calculate minimum detectable effect sizes (S5 Table). We have sufficient power to detect AORs ranging from 1.1 to 2.1, depending on the prevalence of the specific SDoCH. S5 Table describes the minimum detectable odds ratios given the anticipated injury population with examples of low and high prevalence SDoCH.

### Stages of analysis

#### Stage 1: Descriptive statistics

Descriptive statistics for demographic variables (age, sex, geographic location) will be calculated and contingency tables will be generated. Descriptive statistics for injury-related factors (number and type of operative procedures, etiology of the injury, context of injury, intubation at arrival, admission to intensive care unit and ventilator days) will be calculated including measures of central tendency and standard deviations for the injured cohort. Demographics will be compared between cases and the matched controls ensuring similarities between cohorts.

#### Stage 2: Comparison of odds between cohorts

Conditional univariate analyses will be performed on individual SDoCH measures as covariates using case/control as the binary response. A correlation matrix will be calculated to examine overlap between SDoCH categories and guide model development. A conditional multivariable logistic regression model will be calculated including all SDoCH measures to determine odds ratios and adjusted odds ratios using a 95% confidence interval (CI) for cases (injured) and controls (non-injured). A time offset will be incorporated into the model to account for variable pre-injury exposure to the SDoCH. We will stratify analyses by injury severity for subgroup analysis and will create interaction terms and correlation matrices to understand the correlation between sex and SDoCH measures and how this interaction impacts injury risk. The development of these correlation matrices will identify factors that have a stronger association with one sex, informing future research into sex and injury. Given the multiple comparisons (14 SDoCH) we will define statistical significance at p≤.005.

#### Stage 3: Application of a Haddon matrix

Once SDoCH have been identified through statistical modelling, we will determine how these factors fit into the Haddon matrix categories (human, physical environment and socio-economic environment) across the two time points of interest (‘pre-injury’ and ‘injury’). Haddon matrix values (1–9) will be assigned according to interactions in the matrix (factor x time point), and interventions will be conceptualized to reflect each interaction.

## Ethics and dissemination

Study approvals from the Health Information Privacy Committee (HIPC #2017/2018–75), local ethics (H2018-123) and data providers were obtained. As the study will use anonymized whole population administrative data, consent from individual participants is not required. We have created a logic model of our integrated knowledge translation (KT) plan (S6 Table) recognizing that knowledge dissemination will be developed according to findings and intervention points identified by the Haddon matrix and in collaboration with our team. This plan has involved knowledge users (clinicians and collaborators) from the creation of the proposal and will continue to the dissemination of findings. Through this research we will identify key interest groups to engage in creating future prevention programs.

## Supporting information

S1 Table Conceptual biopsychosocial framework specific to pediatric injury, social determinants of child health (SDoCH) and development of subsequent poor health. (DOCX)

S2 Table Haddon matrix of injury prevention. (DOCX)

S3 Table Administrative datasets to be used in the study. (DOCX)

S4 Table ICD-9-CM and ICD-10-CA codes. (DOCX)

S5 Table Minimum detectable effect size (odds ratios). (DOCX)

S6 Table Logic model of research project and integrated knowledge translation plan. (DOCX)

S1 File References for appendices. (DOCX)
