# Supplementary material for: Investigating social determinants of child health and their implications in reducing pediatric traumatic injury: A framework and 17-year retrospective case-control study protocol
Source: PLoS One. 2023 Nov 27;18(11):e0294734. doi: 10.1371/journal.pone.0294734 (PMC10681167; doi:10.1371/journal.pone.0294734)
Supplement: S1 Table — (DOCX) [file pone.0294734.s001.docx]

**S1 Table.** **Conceptual biopsychosocial framework specific to pediatric injury, social determinants of child health (SDoCH) and development of subsequent poor health.**

|  | Biological | Psychological | Social |
| --- | --- | --- | --- |
| *Pre-injury | Sex[1,2]  Physical disorder (mother)[3] | Mental disorder (mother)[4,5] | Gender[1,2]  Socioeconomic status[6]  Income assistance[6]  Urban/rural status[7]  Parental involvement with legal system[8]  Parental education[9]  Children in care[6]  Immigration status[10]  High residential mobility[11]  Age of mother[6,12] |
| *Injury | Sex[1,2]  Type and severity of injury  Age at injury | Mental disorder (mother)[4,5] | Gender[1,2]  Socioeconomic status[6]  Income assistance[6]  Urban/rural status[7]  Parental interaction with the legal system[8]  Parental education[9]  Children in care[6]  Immigration status[10]  High residential mobility[11]  Age of mother[6,12] |
| Post-injury | Scarring and  associated pain[13]  Hypermetabolic/catabolic stress response[14]  Poor subsequent health resulting from injury[15]  Enduring negative effects on growth and development[15] | Post-traumatic  adjustment[16]  Mental disorder (mother)[4,5] | Gender[1,2]  Socioeconomic status[6]  Income assistance[6]  Urban/rural status[7]  Parental interaction with the legal system[8]  Parental education[9]  Children in care[6]  Immigration status[10]  High residential mobility[11]  Age of mother[6,12]  Altered social integration[17]  Interactions with the legal system[8] |

*For this proposal, we are focusing on the ‘pre-injury’ and ‘injury’ periods.

Biological

A hypermetabolic/catabolic stress response occurs with severe injury and lasts for about one-year post-injury.[18] This stress response is characterized by a number of events that are life-threatening to the patient (e.g., tachycardia and cardiac dysfunction, increased cardiac output, increased resting energy expenditure, increased protein and lipid breakdown and peripheral protein wasting). Injuries have also been shown to alter hepatic function by increasing insulin resistance[15] and liver size[19] which may impact patient morbidity and mortality. Pediatric injury survivors may suffer from diminished physical growth and development. The associated weakness and reduced formation of bone growth may increase the risk of fractures later in life.[20]

Psychological

Consequently, the psychosocial aspect for these patients coping with their injuries has become more important.[21] Post-traumatic stress disorder (PTSD) is a common consequence of physical trauma,[22] but a range of other mental disorders can also occur post-injury such as depression, anxiety, panic disorder, generalized anxiety disorder and sleep disorders.[5,23]

Social

Behavioral problems[24] and negative social consequences such as problems returning to school have been reported in younger individuals.[17] This interference with integration into society may have adverse consequences in terms of completion of schooling and future employment leading to an impact on socioeconomic status.
