# Supplementary material for: Investigating social determinants of child health and their implications in reducing pediatric traumatic injury: A framework and 17-year retrospective case-control study protocol
Source: PLoS One. 2023 Nov 27;18(11):e0294734. doi: 10.1371/journal.pone.0294734 (PMC10681167; doi:10.1371/journal.pone.0294734)
Supplement: S2 Table — (DOCX) [file pone.0294734.s002.docx]

**S2 Table. Haddon matrix of injury prevention.**

The Haddon matrix is a validated model used to identify sources of preventable injuries and potential intervention points for injury prevention.[25] Combined with the biopsychosocial framework, it will provide a unique perspective on Social Determinants of Child Health (SDoCH) and their role in not only child traumatic injury risk, but injury prevention and health promotion.

An example of how the Haddon matrix has been applied in the identification of burn injury prevention populations[6] is provided below:

**Haddon matrix for burn injury**

|  | Human | Physical Environment | Socio-economic Environment |
| --- | --- | --- | --- |
| Pre-burn | 1 | 2 | 3 |
| Burn | 4 | 5 | 6 |
| Post-burn | 7 | 8 | 9 |

| Interventions | Targeted population | Haddon matrix | Active / Passive |
| --- | --- | --- | --- |
| Basic burn prevention and first aid education for thermal injuries as part of “Families First” home visits for well babies | Teen mother; Low income; Income assistance | 1, 2, 4, 5, 7 | Active |
| Anti-scald mixing valve subsidized cost for low income families | Low income; Income assistance | 2, 3, 5 | Passive |
| Education for foster parents in burn prevention, first aid and home safety | Child in care | 1, 2, 3, 4, 5 | Active |
| Positive Parenting Program for new parents | Teen mother | 1, 2, 4, 5 | Active |
| Expanded child care supports | Teen mother | 1, 4, 7 | Active |
| Legislation surrounding sources of flame or fire to be out of reach of children | Low income; Income assistance; Child in care; Teen mother | 1, 2 | Passive |
| Expanded rehabilitation, school re-integration and risk avoidance programs | Low income; Income assistance; Child in care; Teen mother | 7, 8, 9 | Active |
