# Supplementary material for: Investigating social determinants of child health and their implications in reducing pediatric traumatic injury: A framework and 17-year retrospective case-control study protocol
Source: PLoS One. 2023 Nov 27;18(11):e0294734. doi: 10.1371/journal.pone.0294734 (PMC10681167; doi:10.1371/journal.pone.0294734)
Supplement: S3 Table — (DOCX) [file pone.0294734.s003.docx]

**S3 Table. Administrative datasets to be used in the study.**

| Database | Contents | Use for this proposed study |
| --- | --- | --- |
| Pediatric Trauma Registry | Demographics, injury-specific details | Identification of individuals with trauma and the clinical factors that may influence the outcomes of interest. |
| Manitoba Health Registry | Scrambled PHIN (sPHIN) for linkage, identify parents | Identification of pediatric trauma patients and their parents. |
| Hospital Separations Abstracts | Admission and discharge date, diagnoses | Identification and measurement of outcomes of interest (hospitalizations for health disorders). |
| Medical Claims (Physician Billings) | Service dates | Identification and measurement of outcomes of interest (physician visits for health disorders). |
| Vital Statistics Mortality | Alive or deceased during study period | Identification and measurement of outcomes of interest (death and cause of death). |
| Prosecutions Information Management System (PIMS) | Incident data/types and involvement | Identification of individuals who have been involved in the justice system |
| Employment / Income Assistance (SAMIN) | Receipt of income assistance | Identification of individuals who have received income assistance |
| Child and Family Services: Applications and Intake | Child in care | Identification of children in care |
| Social Housing Tenant Management System (TMSI) | Residents of social housing units | Identification of households living in social housing units managed by Manitoba Housing |
| Enrollment, Marks, and Assessments (STS/ICAB) | Education performance | Identification of school performance |
| Canada Census | Immigration status* | Identification of children of parent(s) not native to Canada |

⭑ Research Extracts which include the Permanent Resident Database provided by Immigration, Refugees and Citizenship Canada (IRCC)
