# Supplementary material for: Investigating social determinants of child health and their implications in reducing pediatric traumatic injury: A framework and 17-year retrospective case-control study protocol
Source: PLoS One. 2023 Nov 27;18(11):e0294734. doi: 10.1371/journal.pone.0294734 (PMC10681167; doi:10.1371/journal.pone.0294734)
Supplement: S4 Table — (DOCX) [file pone.0294734.s004.docx]

**S4 Table. ICD-9-CM and ICD-10-CA codes.**

| Disorders | ICD-9-CM and ICD-10-CA Codes |
| --- | --- |
| Axis I mental disorders | Anxiety (ICD-9-CM 300.0, 300.2, 300.3; ICD-10-CA F40, F41.0, F41.1, F41.3, F41.8, F41.9, F42, F43.1)  Depression (ICD-9-CM 296.2, 296.3, 296.5,300.4, 309, 311; ICD-10-CA F31.3-F31.5, F32, F33, F34.1, F38.0, F38.1, F43.2, F43.8, F53.0)  Substance use disorders (ICD-9-CM 291, 292, 304, 305, 303; ICD-10-CA F10-F19, F55) |
| Axis II mental disorders | ICD-9-CM 295 to 299.14 (schizophrenic disorders, episodic mood disorders, delusional disorders, other nonorganic psychoses, pervasive developmental disorders) |
| Physical disorders | Cardiovascular disease (ICD-9-CA: 410-414; ICD-10-CM: I20-I25)  Cancer (ICD-9-CA: 140-208; ICD-10-CM: C00.0-C41.9, C45.0-C97)  Chronic obstructive pulmonary disease (COPD) (ICD-9-CA: 491, 492, 494, 496; ICD-10-CM: J41, J42, J43, J44, J47)  Diabetes (ICD-9-CA: 250; ICD-10-CM: E10-E14)  Hypertension (ICD-9-CA: 401-405; ICD-10-CM: I10-I13, I15) |
