# Supplementary material for: Investigating social determinants of child health and their implications in reducing pediatric traumatic injury: A framework and 17-year retrospective case-control study protocol
Source: PLoS One. 2023 Nov 27;18(11):e0294734. doi: 10.1371/journal.pone.0294734 (PMC10681167; doi:10.1371/journal.pone.0294734)
Supplement: S5 Table — (DOCX) [file pone.0294734.s005.docx]

**S5 Table. Minimum detectable effect size (odds ratios).***

| Overall prevalence of exposure* | 0.01 | 0.05 | 0.1 | 0.25 |
| --- | --- | --- | --- | --- |
| Power = 80% | (e.g. child Axis II) | (e.g. immigrant) | (e.g. Maternal axis II) | (e.g. Maternal Axis I) |
| Major injury (n=1000; 5000 controls) | 2.08 | 1.45 | 1.32 | 1.24 |
| Minor injury (n=10,000; 50,000 controls) | 1.3 | 1.14 | 1.1 | 1.08 |
| Power = 90% | | | | |
| Major injury (n=1000; 5000 controls) | 2.32 | 1.54 | 1.38 | 1.29 |
| Minor injury (n=10,000; 50,000 controls) | 1.36 | 1.16 | 1.11 | 1.09 |

*based on estimates from Padalko et al., 2020
