# Supplementary material for: Investigating social determinants of child health and their implications in reducing pediatric traumatic injury: A framework and 17-year retrospective case-control study protocol
Source: PLoS One. 2023 Nov 27;18(11):e0294734. doi: 10.1371/journal.pone.0294734 (PMC10681167; doi:10.1371/journal.pone.0294734)
Supplement: S6 Table — (DOCX) [file pone.0294734.s006.docx]

**S6 Table. Logic model of research project and integrated knowledge translation plan.**

**Goal:** To evaluate the effects of a broad range of Social Determinants of Child Health (SDoCH) on physical traumatic injury risk in children to inform child injury prevention and interventions.

| **Situation** | **Priorities** | **Inputs** | **Outputs** | |  |  |
| --- | --- | --- | --- | --- | --- | --- |
| (1) Research is lacking to support child injury prevention programs due to the inability to understand mechanisms, causes, and risk factors associated with child injury.  (2) Lack of research due to inability to link databases including mechanisms, causes, risk factors, and outcomes associated with child traumatic physical injury.  (3) We need to examine the Canadian context to determine which social and environmental factors place Canadian children at increased risk for traumatic injury so this information can be used to direct decision makers and health policy. | (1) Establish a multidisciplinary team that includes researchers, clinicians and First Nations health experts.  (2) Build clinical, research and partner collaboration and involvement throughout the research process.  (3) Create culturally appropriate research questions, project design and methodology based on identified need.  (4) Consideration of *sex and gender* in each component of the research program.  (5) Create robust research methodology that will produce strong evidence. | ***Resources***  *(What we invest)* | ***Activities***  *(What we will do)* | ***Participants***  *(Who we reach)* | ***Short-Term***  *(1 years)* | ***Future Goals*** |
|  |  | (1) Leaders with clinical expertise in the latest trauma care and research on injury outcomes and sequelae.  (2) Experts in epidemiologic data analysis for health policy.  (3) Infrastructure and staff to support research goals.  (4) First Nations partnership to direct children in care and future investigations into First Nations child health and injury. | (1) Generate findings that will direct decision making and the creation of targeted child injury prevention programs.  (2) Production of widely accessible open-access peer-reviewed publications, presentations, conferences, benchmark reports designed for decision makers and stakeholders in multiple areas:  - Pediatric injury and SDoCH  - Pediatric injury and sex  - Pediatric injury and vulnerable populations  - Injury prevention.  (3) Provide a high-quality training environment and mentorship for early investigators, clinicians and researchers.  (4) Disseminate research findings (presentations, publications, benchmark reports and conferences).  (5) Continue building partnerships with First Nations groups to prioritize First Nations health goals and needs. | General public  Community, including remote and rural centers, clinicians and multidisciplinary teams  Academic health centers  Researchers  Agencies and organizations with interest in First Nations health  Decision makers  Clinical populations: patients, injury survivors, caregivers, family members | (1) Ensure research has met identified goal.  (2) Obtain feedback and input from our research team, partners, patients, families.  (3) Generate future research questions, i.e., future possible studies include both qualitative and quantitative studies.    (4) Explore impact of gender roles on types of child traumatic injury.    (5) Explore the needs of children in care.    (6) Exploring the health needs and injury risk of First Nations children. | (1) Creation of child injury prevention programs targeted at those individuals and times of greatest vulnerability.  (2) Reduced rates of child traumatic injury.  (3) Improved child health and safety.  (4) Safe environments for all children and youth. |
