# Supplementary material for: Investigating social determinants of child health and their implications in reducing pediatric traumatic injury: A framework and 17-year retrospective case-control study protocol
Source: PLoS One. 2023 Nov 27;18(11):e0294734. doi: 10.1371/journal.pone.0294734 (PMC10681167; doi:10.1371/journal.pone.0294734)
Supplement: S1 File — (DOCX) [file pone.0294734.s007.docx]

**S7 File. References for appendices.**

1. Morrongiello BA, Dawber T. Mothers’ responses to sons and daughters engaging in injury-risk behaviors on a playground: Implications for sex differences in injury rates. J Exp Child Psychol [Internet]. 2000 Jun 1;76(2):89–103. Available from: https://pubmed.ncbi.nlm.nih.gov/10788304/

2. Canadian Paediatric Society, Yanchar NL, Warda LJ, Fuselli P. Child and youth injury prevention: A public health approach. Paediatrics and Child Health (Canada) [Internet]. 2020 [cited 2020 Sep 20]. Available from: https://www.cps.ca/documents/position/child-and-youth-injury-prevention#ref15

3. Chen C. Effects of parental chronic illness on children’s psychosocial and educational functioning: A literature review. Contemp Sch Psychol [Internet]. 2017 Jun 22;21(2):166–76. Available from: https://link.springer.com/article/10.1007/s40688-016-0109-7

4. Nevriana A, Pierce M, Dalman C, Wicks S, Hasselberg M, Hope H, et al. Association between maternal and paternal mental illness and risk of injuries in children and adolescents: Nationwide register-based cohort study in Sweden. BMJ [Internet]. 2020 Apr 8;369:m853. Available from: https://www.bmj.com/content/369/bmj.m853

5. Enns J, Gawaziuk JP, Khan S, Chateau D, Bolton JM, Sareen J, et al. Mental and physical health outcomes in parents of children with burn injuries as compared with matched controls. Journal of Burn Care & Research [Internet]. 2016 Jan;37:e18–26. Available from: https://pubmed.ncbi.nlm.nih.gov/26361326/

6. Padalko AA, Gawaziuk J, Logsetty S, Chateau D, Sareen J, Logsetty S. Social determinants associated with pediatric burn injury: A population based, case-control study. Journal of Burn Care & Research. 2020 Mar;41(Supplement_1):S210–1.

7. Bancej C, Arbuckle TE. Injuries in Ontario farm children: A population based study. Injury Prevention [Internet]. 2000 Jun;6(2):135–40. Available from: https://pubmed.ncbi.nlm.nih.gov/10875671/

8. Akesson B, Smyth JMG, Mandell DJ, Doan T, Donina K, Hoven CW. Parental involvement with the criminal justice system and the effects on their children: A collaborative model for researching vulnerable families. Soc Work Public Health [Internet]. 2012;27(1–2):148–64. Available from: https://pubmed.ncbi.nlm.nih.gov/22239383/

9. Beiki O, Karimi N, Mohammadi R. Parental educational level and injury incidence and mortality among foreign-born children: a cohort study with 46 years follow-up. J Inj Violence Res [Internet]. 2013 Jul 1;6(1):37. Available from: https://www.ncbi.nlm.nih.gov/pmc/articles/PMC3865454/

10. Saunders NR, Macpherson A, Guan J, Guttmann A. Unintentional injuries among refugee and immigrant children and youth in Ontario, Canada: A population based cross-sectional study. Injury Prevention [Internet]. 2018;24(5):337–43. Available from: https://pubmed.ncbi.nlm.nih.gov/28951486/

11. Hutchings HA, Evans A, Barnes P, Demmler JC, Heaven M, Healy MA, et al. Residential moving and preventable hospitalizations. Pediatrics [Internet]. 2016 Jul 1;138(1). Available from: https://pubmed.ncbi.nlm.nih.gov/27260695

12. Orton E, Kendrick D, West J, Tata LJ. Independent risk factors for injury in pre-school children: Three population-based nested case-control studies using routine primary care data. PLoS One [Internet]. 2012 Apr;7(4):e35193. Available from: https://pubmed.ncbi.nlm.nih.gov/22496906/

13. Stone J, Gawaziuk JP, Khan S, Chateau D, Bolton JM, Sareen J, et al. Outcomes in adult survivors of childhood burn injuries as compared with matched controls. Journal of Burn Care and Research [Internet]. 2016;37(2):e166–73. Available from: https://pubmed.ncbi.nlm.nih.gov/26594866/

14. Gauglitz GG, Herndon DN, Kulp GA, Meyer  3rd WJ, Jeschke MG. Abnormal insulin sensitivity persists up to three years in pediatric patients post-burn. J Clin Endocrinol Metab [Internet]. 2009;94(5):1656–64. Available from: https://pubmed.ncbi.nlm.nih.gov/19240154/

15. Auger C, Samadi O, Jeschke MG. The biochemical alterations underlying post-burn hypermetabolism. Biochim Biophys Acta Mol Basis Dis [Internet]. 2017 Oct;1863(10):2633–44. Available from: https://pubmed.ncbi.nlm.nih.gov/28219767/

16. Wise AE, Delahanty DL. Parental factors associated with child post-traumatic stress following injury: A consideration of intervention targets. Frontiers in Psychology [Internet]. 2017;8:1412. Available from: https://pubmed.ncbi.nlm.nih.gov/28878711/

17. Han PP, Holbrook TL, Sise MJ, Sack DI, Sise CB, Hoyt DB, et al. Postinjury depression is a serious complication in adolescents after major trauma: Injury severity and injury-event factors predict depression and long-term quality of life deficits. Journal of Trauma - Injury, Infection and Critical Care [Internet]. 2011 Apr;70(4):923–30. Available from: https://pubmed.ncbi.nlm.nih.gov/21610397/

18. Mayou RA, Ehlers A, Bryant B. Posttraumatic stress disorder after motor vehicle accidents: 3-year follow-up of a prospective longitudinal study. Behaviour Research and Therapy [Internet]. 2002 Jun 1;40(6):665–75. Available from: https://pubmed.ncbi.nlm.nih.gov/12051485/

19. Jeschke MG, Gauglitz GG, Kulp GA, Finnerty CC, Williams FN, Kraft R, et al. Long-term persistence of the pathophysiologic response to severe burn injury. PLoS One [Internet]. 2011 Jan;6(7):e21245. Available from: https://pubmed.ncbi.nlm.nih.gov/21789167/

20. Prasad V, West J, Sayal K, Kendrick D. Injury among children and young people with and without attention-deficit hyperactivity disorder in the community: The risk of fractures, thermal injuries, and poisonings. Child Care Health Dev [Internet]. 2018 Nov 1;44(6):871–8. Available from: http://doi.wiley.com/10.1111/cch.12591

21. Zatzick D, Jurkovich GJ, Rivara FP, Wang J, Fan MYY, Joesch J, et al. A national US study of posttraumatic stress disorder, depression, and work and functional outcomes after hospitalization for traumatic injury. Ann Surg [Internet]. 2008 Sep;248(3):429–37. Available from: https://pubmed.ncbi.nlm.nih.gov/18791363/

22. Zatzick D, Roy-Byrne P, Russo J, Rivara F, Droesch RA, Wagner A, et al. A randomized effectiveness trial of stepped collaborative care for acutely injured trauma survivors. Arch Gen Psychiatry [Internet]. 2004 May;61(5):498–506. Available from: https://pubmed.ncbi.nlm.nih.gov/15123495/

23. Hart T, Hoffman JM, Pretz C, Kennedy R, Clark AN, Brenner LA. A longitudinal study of major and minor depression following traumatic brain injury. Arch Phys Med Rehabil [Internet]. 2012 Aug;93(8):1343–9. Available from: https://pubmed.ncbi.nlm.nih.gov/22840833/

24. Roberts NP, Kitchiner NJ, Kenardy J, Bisson JI. Early psychological interventions to treat acute traumatic stress symptoms. Cochrane Database of Systematic Reviews [Internet]. 2010 Mar 17. Available from: https://www.cochranelibrary.com/cdsr/doi/10.1002/14651858.CD007944.pub2/full

25. World Health Organization. Unintentional Childhood Injuiries [Internet]. 2015 [cited 2022 Jun 1]. Available from: https://slideplayer.com/slide/4907196/16/images/1/Children+s+Health+and+the+Environment.jpg
